# Heparin-induced thrombocytopenia presenting as splenic hemorrhage following cardiac surgery: a case report

**DOI:** 10.1186/s12959-021-00257-y

**Published:** 2021-01-19

**Authors:** Joseph Ferry, Samuel Youssef, Pierce Wu, Livia Hegerova

**Affiliations:** 1 Department of Hematology and Oncology, Swedish Medical Center, 747 Broadway 12th Fl. East Tower, Seattle, WA 98122 USA; 2 Swedish Heart and Vascular – Cardiac Surgery, Seattle, WA USA; 3 Swedish Center for Blood Disorders and Stem Cell Transplantation, Seattle, WA USA

**Keywords:** Bilateral adrenal hemorrhage, Heparin, Splenic infarction, Thrombocytopenia, Thrombosis

## Abstract

**Background:**

Heparin-induced thrombocytopenia with thrombosis (HITT) is a paradoxical prothrombotic complication of anticoagulant therapy. As many as 3% of patients undergoing cardiac surgery develop clinical HIT presenting as thrombocytopenia with or without thrombosis within 5–10 days of heparin exposure. Thrombotic complications associated with HIT carry a mortality rate of 5–10%.

**Case presentation:**

We report a case of atraumatic splenic hemorrhage due to splenic vein thrombosis as the main indicator of HIT following cardiac surgery in a 62-year-old woman. She presented to the emergency department on day nine following coronary artery bypass graft surgery with acute weakness, dizziness, and malaise. Her evaluation in the emergency department found anemia and thrombocytopenia. A coagulation profile revealed a markedly elevated d-dimer. She underwent a computed tomography scan of the chest, abdomen and pelvis for suspected bleed and was found to have splenic vein thrombosis, right atrial filling defects consistent with atrial thrombus and mild to moderate hemoperitoneum. Surgical consultation was obtained due to splenic hemorrhage. Hematology was consulted on post-operative day 10, however, she unfortunately developed left sided weakness concerning for stroke. A magnetic resonance imaging scan of the brain demonstrated infarct involving distribution of the right anterior cerebral artery. A transesophageal echocardiogram demonstrated a large immobile thrombus within the right atrium with a second, mobile thrombus arising from the left tricuspid valve annulus. Due to a 4Ts score of 7 and markedly positive platelet factor 4 (PF4) IgG antibody a serotonin release assay was not performed given the high probability of HIT. She was cautiously treated with bivalirudin and was transitioned to warfarin anticoagulation. In the following days her platelet count recovered and 3 months later a transthoracic echocardiogram revealed solution of the intracardiac thrombi.

**Conclusions:**

Atraumatic splenic hemorrhage is an unusual presentation of HIT that is reminiscent of the rare bilateral adrenal hemorrhage due to adrenal necrosis that also occurs in HIT. Alternative anticoagulation is the mainstay of therapy for HIT despite hemorrhage, given the underlying acquired hypercoagulability. Despite similarities of the presentation between splenic hemorrhage and bilateral adrenal hemorrhage, splenic hemorrhage is rarely described in the literature. HIT should be considered in patients presenting with thrombocytopenia following cardiac surgery.

## Essentials

- HIT-associated antibodies are frequently detected after cardiac surgery
- We report a case of atraumatic splenic hemorrhage due to HIT following coronary artery bypass grafting.
- HIT should be suspected for any patient with congestive hemorrhage following venous thrombosis, thrombocytopenia, and recent heparin exposure
- Hemorrhagic splenic infarction due to splenic vein thrombosis is reminiscent of bilateral adrenal hemorrhage (BAH) due to adrenal necrosis seen in HIT

## Background

Heparin-induced thrombocytopenia (HIT) is a complication of heparin therapy which occurs more commonly in postoperative than medical patients [1]. Up to 50% of patients undergoing cardiac surgery develop antibodies to platelet factor 4 (PF4) complexed to heparin, attributed to platelet activation and high heparin doses during cardiopulmonary bypass [2]. Only 1 to 3% of these patients will develop clinical HIT manifesting with thrombocytopenia with or without thrombosis [2, 3]. HIT typically presents 5–10 days following exposure to heparin, often with platelet decline greater than 50% of baseline and is associated with life and limb-threatening thrombotic complications. HIT should be suspected in any patient who has received heparin and develops thrombocytopenia, given high mortality (5–10%) typically a result of thrombotic complications [1].

One increasingly recognized and unusual thrombotic complication that is seen in HIT is adrenal vein thrombosis with tissue necrosis leading to bilateral adrenal hemorrhage (BAH). This congestive hemorrhage following venous thrombosis should raise high clinical suspicion for HIT. We present a case of splenic vein thrombosis post-cardiac surgery complicated hemorrhagic infarct, reminiscent of that seen in BAH, that led to the diagnosis of HIT.

## Case presentation

A 62-year-old woman with a history of congestive heart failure, coronary artery disease, diabetes mellitus type 2, and recent coronary artery bypass graft (CABG) presented to the emergency department with acute weakness, dizziness, and malaise.

Past surgery history was significant for a two-vessel coronary artery bypass graft involving the left internal mammary artery to the left anterior descending artery and the left radial to obtuse marginal artery nine days prior to presentation. In the perioperative period she received three heparin boluses at 40u/kg based on a weight of 66.7 kg as well as a heparin infusion dosed between 16u/kg/hr-26u/kg/hr. for 3 days. She had no family history of hematological disorders or hypercoagulability. She was on low-dose aspirin. Her complete blood count (CBC) was normal prior to CABG including platelet count of 268 x 10P^3^P/μL (normal 150–379 x 10P^3^P/μL) and at hospital discharge was 174 x10P^3^P/μL with post-operative hematocrit of 36% (normal 37.5–51%).

On physical examination she was afebrile, hypotensive with systolic blood pressure in the 70s, and in rapid atrial fibrillation with heart rate 120–130 s. She denied abdominal pain and had a benign abdominal examination. Laboratory data revealed white blood cell count 10.7 x 10P^9/^PL (normal 3.4–10.8 x 10P^9/^PL), hematocrit 19%, and platelet count of 49 x10P^3^P/μL. Complete metabolic panel and lactate were normal. Troponin was elevated at 0.21 ng/mL (normal 0.00–0.07 ng/mL. Chest x-ray was normal. A limited beside echocardiogram demonstrated no pericardial effusion. She received intravenous fluid resuscitation and transferred to the intensive care unit for further evaluation.

Upon arrival to the intensive care unit, she received 2 units of packed red blood cells with appropriate increase to hematocrit 25% post-transfusion and 1 unit of platelets with increase to 73 x10P^3^P/μL. Further laboratory evaluation was negative for hemolysis with normal lactate dehydrogenase 292 U/L (normal 119–226 U/L) and haptoglobin 98 mg/dL (normal 34–200 mg/dL). A disseminated intravascular coagulopathy (DIC) panel demonstrated d-dimer of > 40.00 μg/ml (normal <=0.49 μg/ml), normal fibrinogen 214 mg/dL (normal 175–475 mg/dL), international normalized ratio (INR) 1.4 (normal 0.9–1.1), protime (PT) 16.7 s (normal 12.0–14.4 s), partial thromboplastin time (PTT 37.3 s (normal 22.0–35.0 s), normal thrombin time (TT), and normal red blood cell morphology.

To evaluate for bleeding, a computed tomography (CT) angiogram of the chest, abdomen, and pelvis was ordered and demonstrated filling defects suggestive of right atrial thrombus, splenic vein.

thrombosis with splenic infarct, and mild to moderate associated hemoperitoneum. She denied abdominal trauma. Surgery service was consulted and concerned about splenic hemorrhage. Hematology was consulted to evaluate the etiology of spontaneous splenic vein thrombosis with associated hemorrhagic infarction.

**Fig. 1:**
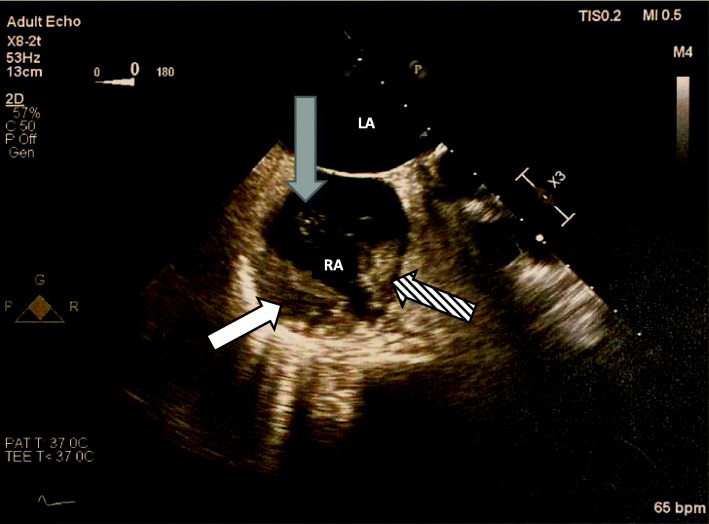
Transesophageal Echocardiogram (TEE). White arrow demonstrating immobile right atrial thrombus. Striped arrow demonstrates second mobile thrombus. Grey arrow demonstrates thrombin strands originating from mobile thrombus

Unfortunately, on the day of Hematology evaluation (post-operative day 10) she developed left-sided weakness and balance changes concerning for stroke and an MRI revealed infarct involving the distribution of the right anterior cerebral artery. A transesophageal echocardiogram revealed large immobile thrombus in the right atrium with a second thrombus adjacent to this which was mobile and.

arising from the left tricuspid valve annulus, large thrombus in the left atrial appendage, and absence of a patent foramen ovale (Fig. 1). She was not a candidate for thrombolytic therapy due to thrombocytopenia. She was calculated to have a 4Ts score of 7. A heparin PF4 IgG antibody was.

markedly positive with 3.290 OD. Serotonin release assay was not performed due to the high probability of HIT evidenced by the markedly positive PF4 IgG and 4Ts score of 7. Together these predictive tools indicate a 72% likelihood ratio and 99% posttest probability of HIT [4]. Lower extremity ultrasound was negative for thrombosis while upper extremity ultrasound showed cephalic vein thrombosis. The patient was diagnosed with heparin-induced thrombocytopenia with thrombosis (HITT).

Given extensive thrombosis in HIT, anticoagulation was cautiously started with bivalirudin. Our organization utilizes direct thrombin inhibitor (DTI) levels to monitor and adjust bivalrudin dosing. Given the presence of hemoperitoneum prior to bivalruin dosing, bivalrudin was started at 10.01 mg/hr. and titrated to 12.012 mg/hr. based on DTI levels and a dose of 0.13–0.156 mg/kg/hr. at a weight of 77 kg. DTI levels were maintained at goal between 60 and 90 s throughout therapy. She remained clinically stable from the intrabdominal hemorrhage and transitioned to warfarin anticoagulation. Platelets recovered to 108 x10P^3^P/μL four days after starting alternative anticoagulation and greater than 150 x10P^3^P/μL nine days after therapy for HITT initiated. The patient had repeat transthoracic echocardiogram after 3 months of warfarin anticoagulation with the resolution of intra-cardiac thrombi. She continues on long-term anticoagulation for atrial fibrillation.

## Discussion and conclusions

Heparin-induced thrombocytopenia (HIT) is a paradoxical prothrombotic syndrome resulting from exposure to heparin-containing products [5–8]. HIT-associated antibodies are frequently detected after cardiac surgery [9]. Thrombosis in the setting of HIT is more commonly associated with the venous system, however, arterial thrombus formation is more common in patients receiving heparin for cardiovascular disease [5]. We report a case of atraumatic splenic hemorrhage due to splenic vein infarction as the main indicator of the diagnosis of HIT. The presentation is reminiscent of the rare bilateral adrenal hemorrhage due to adrenal vein thrombosis that has been reported in HIT. Given the high mortality of HIT, as high as 28% in cardiac surgery patients, awareness of these unique presentations is critical for earlier intervention [10].

HIT should be suspected in any patient who has had prior heparin exposure who presents with thrombocytopenia, especially if the fall is greater than 50% of baseline count and whether the nadir remains within the normal range [6]. Our patient had a high 4 T score and HIT PF4 antibody OD of 3.2 and new splenic and atrial thrombosis, confirming a diagnosis of HITT. Anticoagulation was started with parenteral direct thrombin inhibitor and transitioned to warfarin once platelet count was within the normal limit for 2 consecutive days. Alternative anticoagulation is the mainstay of therapy for HITT even in the setting of hemorrhage, given the underlying acquired hypercoagulability.

A well-documented and increasingly well-recognized thrombotic complication of HIT is bilateral adrenal hemorrhage [7]. Clues to this rare complication include the development of abdominal pain, diarrhea, and fever which often lead to imaging of the abdomen and revelation of injury to the adrenal glands and is confirmed by corticotropin-stimulation test [7, 11]. The risk of BAH in a case study of HIT by *Warkentin et. al* was 1.6% [12]. Strong arterial blood supply with a single central vein makes the.

adrenal glands susceptible to congestive hemorrhage following venous thrombosis [13]. We hypothesize that the spleen is at similar risk given it too has a rich arterial supply and single splenic vein.

A literature review reveals few reported cases of splenic vein thrombosis with hemorrhagic infarction as a clinical sequela of HIT. Earlier reports discussed spontaneous splenic ruptures occurring on heparin anticoagulants, however did not report on HIT antibody testing [14–17]. One case report had a very similar presentation of splenic hematoma after cardiac surgery, with splenectomy pathology showing multiple fibrin thrombi in the spleen [9]. This pathology confirms our hypothesis of microthrombi leading to subsequent hemorrhagic conversion in the spleen, as occurs in BAH.

Early imaging to identify these unusual intra-abdominal thrombotic complications of HIT is encouraged [18]. A case report by *Lammering* et al. discusses a case of splenic rupture secondary to splenic vein thrombosis resulting from HIT, while the discussion focuses on the utility of multidetector-.

row computed tomography in the rapid and accurate diagnosis of HIT related thromboembolic complication. *Lammering* et al. describe the case in the setting of non-specific abdominal pain which differed from our patient without abdominal pain [19].

Treatment of HIT involves alternative non-heparin anticoagulation in accordance with published guidelines including those by the American College of Chest Physicians and the updated *American Society of Hematology Guidelines for Management of Venous Thromboembolism: Heparin-Induced Thrombocytopenia* [1, 20]. Despite hemorrhagic complications such as BAH, or as in our patient intraabdominal hemorrhage due to splenic infarction, treatment is careful anticoagulation given the underlying etiology for hemorrhage is hypercoagulability. Multiple cases reports on BAH advocate for prompt initiation of appropriate anticoagulation to ensure a successful outcome [12, 21]. We advocate for a similar approach to congestive splenic hemorrhage following splenic vein thrombosis as a consequence of HIT.

This case emphasizes that the diagnosis of HIT should be considered for patients with unusual intra-abdominal thrombosis or hemorrhage, especially within the expected time course following heparin exposure.

Congestive hemorrhage following venous thrombosis, with recent heparin exposure and declining platelet count, should raise clinical suspicion of HIT. The diagnosis of HIT-induced splenic vein thrombosis and subsequent hemorrhagic infarct mimics the rare bilateral adrenal hemorrhage due to adrenal vein thrombosis seen in HIT. Multidetector-row computed tomography should be considered for the evaluation of thromboembolic sequelae in patients with unexplained anemia or abdominal pain with suspicion of HITT to exclude adrenal or splenic involvement. Alternative anticoagulation remains the mainstay of therapy.

## Data Availability

No datasets were developed or analyzed for this case report.

## Abbreviations

BAH: bilateral adrenal hemorrhage
CABG: coronary artery bypass graft
CBC: complete blood count
CT: computed tomography
DIC: disseminated intravascular coagulopathy
HITT: heparin induced thrombocytopenia with thrombosis
HIT: heparin induced thrombocytopenia
PT: protime
PTT: partial thromboplastin time
TT: thrombin time
